# The 14 bp Del/Ins HLA-G Polymorphism Is Related with High Blood Pressure in Acute Coronary Syndrome and Type 2 Diabetes Mellitus

**DOI:** 10.1155/2014/898159

**Published:** 2014-02-06

**Authors:** Ilian Janet García-González, Yeminia Valle, Fernando Rivas, Luis Eduardo Figuera-Villanueva, José Francisco Muñoz-Valle, Hector Enrique Flores-Salinas, Bianca Ethel Gutiérrez-Amavizca, Nory Omayra Dávalos-Rodríguez, Jorge Ramón Padilla-Gutiérrez

**Affiliations:** ^1^Instituto de Investigación en Ciencias Biomédicas, Centro Universitario de Ciencias de la Salud, Universidad de Guadalajara, Sierra Mojada 950, Edificio Q, Primer Piso, Colonia Independencia, 44350 Guadalajara, JAL, Mexico; ^2^Centro Universitario de Ciencias de la Salud, Universidad de Guadalajara, Sierra Mojada 950, Colonia Independencia, 44350 Guadalajara, JAL, Mexico; ^3^Hospital General de Occidente, Secretaria de Salud Jalisco, Av. Zoquipan 1050, Colonia Zoquipan, 45170 Zapopan, JAL, Mexico; ^4^IMSS, Centro Medico Nacional de Occidente, Belisario Dominguez 1000, Colonia Independencia, 44340 Guadalajara, JAL, Mexico; ^5^Instituto de Investigación en Genetica Humana, Centro Universitario de Ciencias de la Salud, Universidad de Guadalajara, Sierra Mojada 950, Edificio P, Segundo Piso, Colonia Independencia, 44350 Guadalajara, JAL, Mexico

## Abstract

Immunologic and inflammatory processes are involved in the pathogenesis of acute coronary syndrome (ACS) and type 2 diabetes mellitus (DM2). Human leukocyte antigen-G (HLA-G) is a negative regulator of the immune response. This study evaluates the 14 bp Del/Ins HLA-G polymorphism in ACS and DM2. Three hundred and seventy individuals from Western Mexico were recruited and categorized into three groups: ACS (86), DM2 without coronary complications (70), and healthy subjects (214). Genotyping of the 14 bp Del/Ins HLA-G polymorphism was performed by PCR and Native-PAGE. The most common risk factors were hypertension and overweight in ACS and DM2, respectively. The genetic distribution of the 14 bp Del/Ins HLA-G polymorphism showed no significant differences between groups (*P* ≥ 0.23). Nonetheless, the Ins/Ins genotype was associated with high blood pressure (HBP) in the DM2 group (OR_c_ = 1.65, *P* = 0.02). The genetic recessive model showed similar findings (OR_c_ = 3.03, *P* = 0.04). No association was found in ACS, with a *P* of 0.05; nevertheless, the prevalence of Ins/Ins carriers was quite similar to that found in the DM2-HBP group. The 14 bp Del/Ins HLA-G polymorphism was not a susceptibility factor for ACS or DM2; however, the Ins/Ins genotype might have contributed to the development of HBP in the studied groups.

## 1. Introduction

Acute coronary syndrome (ACS) is a leading cause of morbidity and mortality around the world [1, 2]. It belongs to the group of clinical entities that have acute myocardial ischemia as a common physiopathological mechanism and includes unstable angina and myocardial infarction with (STEMI) and without (NSTEMI) ST elevation [3]. Acute ischemia is in most cases produced by the rupture of an atheromatous plaque to which platelets are recruited until an occlusive thrombus is formed [4, 5]. Nevertheless, it is increasingly recognized that both immunologic and inflammatory mechanisms participate in the progression and destabilization of atheromatous lesions [6]. It is well known that type 2 diabetes mellitus (DM2) and obesity are the main chronic diseases related with ACS. Besides, various studies demonstrate that an important inflammatory component is also present in both conditions [7–10]. Multiple reports have established an association of the immune response and its genetic component with the pathogenesis of ischemic heart disease [11–13].

The human leukocyte antigen-G (HLA-G) is part of the nonclassical major histocompatibility complex class-I and functions as a negative regulator of the immune response with anti-inflammatory activity [14]. Due to the pathophysiologic mechanisms of cardiovascular diseases, attention has been focused on this molecule, considering its role in immunological and inflammatory pathways and in the inhibition of endothelial cell proliferation [15].

The HLA-G polymorphism (rs66554220) consists in a deletion (Del) or insertion (Ins) of 14 base pairs (bp) in the +2960 position in exon 8. The Ins allele has been associated with an alternative splicing where 92 bp are removed, which changes the stability of the mRNA and diminishes HLA-G levels [14]. This polymorphism and HLA-G soluble levels are associated with different disorders such as recurrent abortion [16–18], autoimmune diseases [19–22], cancer [23–25], and inflammatory diseases, including coronary artery disease (CAD) [13].

Many investigations support the idea that polymorphic variants in genes involved in the endothelial function [26], inflammation [27], lipid metabolism [28], thrombosis [29], and fibrinolysis [30] are strongly associated with ACS. ACS and DM2 have different pathophysiologic mechanisms; however, they share a close relationship and both converge in a chronic inflammatory state involving endothelial dysfunction [31, 32]. The latter has been proposed as the trigger of the progression and complications of cardiac lesions [31, 33]. Some studies have demonstrated that DM2 patients commonly present ACS and that a raised proportion of ACS patients have diabetes as a comorbid condition [34, 35]. Indeed, cardiovascular disease is a leading cause of mortality in diabetic individuals [36, 37]. Since diabetes has been categorized as a low-grade inflammation disease [38], we hypothesize that HLA-G polymorphism has a major role in ACS compared to individuals without cardiovascular history, including the DM2 and healthy subjects (HS) groups.

The purpose of the present work was to analyze 14 bp Del/Ins HLA-G polymorphism in ACS and associated risk factors in 370 individuals. As comparison groups, we included DM2 and healthy subjects.

## 2. Methods

### 2.1. Subjects

We studied 370 individuals with the following characteristics.

1. Two hundred and fourteen HS blood donors were presented. Absence of any chronic (DM2, high blood pressure, HBP, cardiovascular diseases) or infectious diseases is a prerequisite for being a blood donor.
2. A total of 86 patients with acute coronary syndrome (63 males, 23 females) were diagnosed according to the criteria of the American College of Cardiology (ACC) [39]. Their medical record of classical risk factors, defined according to the ACS, was registered and categorized as present or absent.
3. Seventy type 2 diabetes mellitus patients without coronary complications (27 males; 43 females) were classified as stated by the American Diabetes Association (ADA) [40].

All the subjects, age-matched, were recruited from the Hospital de Especialidades del Centro Medico Nacional de Occidente del Instituto Mexicano del Seguro Social (CMNO-IMSS). The mean age was 65 ± 13.55 years.

Only those individuals who for three generations, including their own, had been born in Western Mexico were considered. Mexican mestizo population is an admixture of Amerindian, European, and African components with an estimated contribution of 21–25%, 60–64%, and 15%, respectively [41].

*Ethical Considerations*. The study was made in accordance with the Declaration of Helsinki. All patients and subjects accepted to participate and an informed written consent was obtained. In addition, ethical approval was obtained by the Centro Universitario de Ciencias de la Salud, CUCS, UdeG (C.I. 069-2012).

### 2.2. Genetic Analysis

Genomic DNA (gDNA) was purified from peripheral blood by the salting out method [42]. The rs66554220 polymorphism was amplified by polymerase chain reaction (PCR) using the following primer sequences: forward, 5′-GAT GGG CTG TTT AAA GTG TCA CC-3′ and reverse, 5′-GGA AGG AAT GCA GTT CAG CAT G-3′. PCR amplification was carried out in a total volume of 10 *μ*L containing 10 ng/*μ*L of gDNA, 0.02 U/*μ*L of Taq DNA polymerase (Invitrogen Life Technologies), 1X of buffer, 0.6 pM of each primer, 1.5 mM of MgCl_2_, and 0.1 mM of dNTP. The thermocycling conditions had an initial denaturation step of 4 min at 94°C followed by 28 cycles of 26 s each at 94°C, 65°C, and 72°C and the final extension step of 7 min at 72°C. The amplified PCR products were analyzed by 6% polyacrylamide gels (38 : 2; acrylamide : bis-acrylamide, resp.) stained with silver nitrate. Amplification of the HLA-G Del/Ins polymorphism resulted in 210 bp (Del) or 224 bp (Ins) fragments.

### 2.3. Statistical Analysis

The statistical analysis was carried out using the SPSS statistical package version 20.0, Excel 2010, and Genetic Data Analysis [43]. The *χ* ^2^ or Fisher's exact test, when applicable, was used to compare discrete variables and to test the Hardy-Weinberg equilibrium. The Kolmogorov-Smirnov test and the sample size were considered in order to categorize parametrical (*t*-test, ANOVA) and nonparametrical tests (Spearman correlation, Kruskal-Wallis test, Mann-Whitney *U* test). The data for continuous variables were expressed as means ± standard deviation (SD). The significance level was <0.05. The odds ratio (OR) was the measure of association. Bivariate and multivariate logistic regression analyses were accomplished to adjust the risk for every independent variable and to know the risk of polymorphism with ACS.

## 3. Results

### 3.1. Description of Clinical Variables

*Clinical Data of HS*. All the laboratory test values were within the reference values (Table 1): glucose = 85.53 ± 12.92 ng/dL; triglycerides = 102.50 ± 24.59 ng/dL; cholesterol = 156.09 ± 21.54 ng/dL; high-density lipoprotein = 49 ± 9.99 ng/dL; low-density lipoprotein = 90.30 ± 15.4 ng/dL.

The clinical data of the ACS group is shown in Table 1. The most/least prevalent risk factors in ACS were HBP (55.81%) and chronic obstructive pulmonary disease (9.3%), respectively. The main antiplatelet agent prescribed was aspirin; heparin was prescribed as antithrombotic, while captopril, an angiotensin converting enzyme inhibitor, was selected as *first-line* therapy for the treatment of hypertension and heart failure.

The mean age of disease onset in the DM2 group was 42 years. In average, for each patient there are 1.25 direct blood relatives affected with DM2, while 52.24% and 49.26% of diabetics had overweight and high blood pressure, respectively. The most prevalent complications were ocular (35.82%), with retinopathy accounting for 17.43%, and dental (31.34%). With respect to treatment, 67.16% of patients were receiving glibenclamide as a hypoglycemic agent, and 34.33% were receiving metformin as an antidiabetic.

### 3.2. Genetic Association

The genotype frequencies were in accordance with the Hardy-Weinberg equilibrium, with the exception of those of the DM2 group (HS: *P* = 0.41, ACS: *P* = 0.66 and DM2: *P* = 0.02). The allele frequencies in HS were compared with other populations [44], finding differences between European, Amerindian, and Asiatic populations (*P* ≤ 0.3), but not between the Southeast Asian and African (*P* > 0.21) groups.

The allele and genotype frequencies are depicted in Table 2. There was no significant difference in the genetic distribution of ACS and DM2 when compared to HS (*P* > 0.23). In addition, a similar allele and genotype frequency was observed between disease groups (*P* > 0.09), even when risk factors were stratified (for instance, ACS-without DM2 versus the DM2 group, data not shown).

Additionally, the main risk factors and some demographic characteristics of the disease groups were stratified by genotype (Table 3). The heterozygous genotype was overrepresented in diabetic patients with overweight, renal, retinal, and dental complications. However, these subgroups had a similar genetic distribution. A significant difference was found in DM2 genotype frequencies, where Ins/Ins carriers had 1.65 (*P* = 0.02) more probability to present HBP than wild genotype carriers, 24% versus 17%, respectively. The variables of genotype, age of onset, duration, overweight, and HBP were analyzed by logistic regression, corroborating the significance of the latter. When the model was adjusted according to the genotype, the presence of the homozygous genotype explained an association with HBP (OR corrected or OR_C_ = 1.65, CI 0.14–1.50, *P* = 0.02). This is consistent with the comparisons made by Fisher's exact test in the recessive model (Del/Del + Del/Ins versus Ins/Ins: OR_C_ = 3.03, CI: 0.01–5.8, *P* = 0.04). Regarding the ACS subgroups, a marginal difference was observed in the dominant model between ACS without HTA and ACS-HTA. As can be noticed, some of the confidence intervals are wide; this can be explained by the effect of the size of the sample.

## 4. Discussion

The haemostatic and inflammatory responses participate in the atherosclerotic process, the growth of the lesions, and the onset of atherothrombotic complications. Some characteristics of HLA-G could be highlighted, such as the low polymorphism level when compared to classical molecules, the specific tissue expression, and trogocytosis capacity. In the latter, the cells become temporarily HLA-G positive, acquiring immunosuppressive effects [14]. HLA-G has been extensively studied in the maternal-fetal interface, mainly because of its expression in trophoblastic cells [45]. Furthermore, HLA-G can be expressed by endothelial cells, which are a key in the regulation of the atherosclerosis process. This is thought to result from chronic inflammation and injury to the arterial wall in the peripheral or coronary vascular system [13, 14, 46]. Some studies have shown that sHLA-G1 inhibits endothelial cell proliferation through apoptosis, providing evidence that HLA-G may play an essential role in vascular remodeling [46, 47]. These antiangiogenic properties would be desirable in the capillary environment, where the microvascular channels that form in atherosclerotic plaques develop as a result of neoangiogenesis [31].

In Mexico, in the last decade, myocardial infarction has been the first and second cause of death among people older than 60 years and the general population, respectively [48]. A sedentary life style, DM2, obesity, and HBP are the main risk factors related with this pathology [49, 50]. However, these classical factors explain only half of the cases, suggesting the importance of genetic factors [51]. Endothelial dysfunction is the main cause involved in the pathophysiology of multifactorial inflammatory diseases such as ACS, DM, and hypertension [31, 32]. Because the 14 bp Ins allele is associated with lower levels of HLA-G mRNA and circulating, soluble HLA-G isoforms [52, 53], the adaptive immunity of variant carriers could be biased towards the Th-1 phenotype, increasing acute phase proteins and proinflammatory molecules such as tumor necrosis factor-alpha (TNF-*α*), interleukin-1 (IL-1), and interferon gamma (INF-*γ*), thus exacerbating disease progression and its complications [31, 54, 55].

Due to the biological importance of the 14 bp Del/Ins polymorphism, we attempted to evaluate its association with ACS and contrast it with a DM2 group without coronary complications.

ACS patients were older than 34 years; STEMI accounted for 80.23% and was more frequent in men, as has been described in [56]. The three most frequent ACS risk factors in Western Mexico were HBP, smoking, and DM2 similar to other reports [47, 57, 58]. Data from the Mexican Register of ACS, RENASICA II [56], puts HBP in second place (55%). In our study, the variation of the proportions might be influenced by sample size; however, this issue highlights the fact that the main comorbidities associated with cardiac chronic diseases are similar across the world, and thus our findings are applicable in all geographical regions.

In the DM2 group, the most prevalent comorbidities were obesity and HBP, similar to other reports [59, 60]. The main complications were ocular and dental, contrasting with the results of Liu et al. [61] where cardiovascular events lead the list. However, we need to take into account that this group had no coronary disease history. Retinopathy was the most common ocular complication, as reported by other researchers [62, 63]. Likewise, as was stated above when dealing with the prevalence of ACS risk factors, our limitation is the size of the sample, which might not reflect the real proportion of these events; a cross-sectional study design will be needed to address this matter.

Regarding the allele frequencies of the 14-bp Del/Ins HLA-G polymorphism, a similar genetic distribution was observed between African and Southeast Asian populations [44]. It is important to notice that the Ins allele has the highest frequency in our group, compared with the rest of the populations. These differences can be explained by Western Mexico mestizos' own genetic structure, which highlights the genetic variability between populations.

There is a previous report involving 14-bp Del/Ins HLA-G polymorphism associated with CAD in an Italian population [13]. They found a significant association between the Ins/Ins genotype and CAD (OR = 1.51, *P* = 0.018). In our study, we found no genetic association in either the ACS or the DM2 group. Further studies including a larger sample size and analyses of other polymorphisms in the same gene and HLA cluster are desirable in order to corroborate the lack of genetic association.

HBP was the clinical variable that could be associated with 14 bp Del/Ins polymorphism. The Ins/Ins genotype carriers in the DM2 group were almost twice more susceptible to present HBP than Del/Del carriers. This association remained significant after the variables were adjusted by linear regression. HBP plays an important role in macrovascular complications of DM2; it is a manifestation of endothelial dysfunction [64, 65] involving the insertion of 14 bp polymorphism in HLA-G. To the best of our knowledge, there are no published studies that evaluate this polymorphism in DM2. Eike et al. [66] determined, by genome-wide scan, an association between HLA-G locus and susceptibility to type 1 diabetes. Otherwise, HLA-G contributes to the trend towards the Th2 phenotype [67, 68], acting as a counterbalance to the characteristic inflammatory response of both types of diabetes; however their pathophysiological mechanisms are different. We found a marginal association (*P* = 0.05) in the ACS patients. The prevalence of Ins/Ins carriers is quite similar in the DM2-HBP group, suggesting that a representative enlargement of the study groups could deny or confirm the role of 14 bp polymorphism in HBP. Nonetheless, some of the confidence intervals are wide and we should take into account the effect of the size of the sample as a limitation of the study.

Regarding other reports dealing with the association of this polymorphism with hypertension, we would like to mention the transmission disequilibrium test conducted by Hylenius et al. [69]. They evaluated mother, father, and child, including preeclamptic primiparas, and found the Ins/Ins genotype overrepresented in the offspring, suggesting that mother-child HLA-G genotypes could influence the risk of developing preeclampsia. Preeclampsia is an entity associated with HBP and proteinuria in 2–7% of pregnant women due to an impaired endothelial function that causes a multisystemic syndrome [64, 70]. Previously, Humphrey et al. [71] had not found an association of this polymorphism with preeclampsia and eclampsia. In addition, our results could be partially correlated with Solini et al. findings [72]. In this study, soluble HLA-G levels in DM2 patients were measured, finding increased levels of this molecule; those who were soluble HLA-G positive had higher body mass index, systolic blood pressure, and cholesterol levels. This could reflect the participation of HLA-G in the modification of blood pressure; however, whether this polymorphism increases the susceptibility to hypertension or is a consequence of chronic diseases requires further study. The association of HLA-G with inflammatory diseases has been poorly investigated; higher or lower levels could be beneficial according to the disease. Since they have a wide spectrum of activities, both the gene and the protein might be promising therapeutic targets for the management and control of chronic diseases.

## 5. Conclusion 

The 14 bp Del/Ins polymorphism was not a susceptibility genetic marker for the ACS and DM2 groups without coronary complications. However, the Ins/Ins genotype was associated with hypertension in DM2.

## Figures and Tables

**Table 1:** Demographic and clinical assessments in HS, ACS and DM2.

HS		DM2	
Parameter	Average ± SE		*n* (%)
		Risk factor	
Glucose (mg/dL)	85.53 ± 12.92	Age (years)	58±13.09
Triglycerides (mg/dL)	102.50 ± 24.59	Affected family	1.25
Cholesterol (mg/dL)	156.09 ± 21.54	Overweight	37 (52.85)
HDL (mg/dL)	49.99 ± 9.99	HBP	34 (48.57)
LDL (mg/dL)	90.30 ± 15.4	Ocular complications	24 (34.28)
		Retinopathy	12 (17.14)
		Treatment	
		Glibenclamide	47 (67.14)
		Metformin	24 (34.28)

ACS			
Parameter	Average ± SE		*n* (%)

		Risk factor	
Ratio male/female	2.74	Obesity	12 (13.95)
Age (years)	65 ± 1.46	DM2	38 (44.19)
Systolic blood pressure (mmHg)	122 ± 3.95	DYS	22 (25.58)
Diastolic blood pressure (mmHg)	72 ± 1.98	HBP	48 (55.81)
Glucose (mg/dL)	156 ± 9.12	Smoking	44 (51.16)
Triglycerides (mg/dL)	199 ± 22.5	COPD	8 (9.3)
Cholesterol (mg/dL)	187 ± 39.16	Treatment	
CK (IU/L)	673 ± 109.5	AA	56 (65.12)
CK-MB (IU/L)	93 ± 14.37	ACE inhibitors	54 (62.79)
Troponin T (ng/mL)	36.38 ± 35.56	AnA	86 (100)
		Antidiabetics	3 (3.49)
		ARB	2 (2.33)
		CCB	10 (11.63)

AA: antithrombin agents; ACE: angiotensin converting enzyme; AnA: antiplatelet agents; ARB: angiotensin II receptor blockers; CCB: calcium channel blockers; CK: creatine phosphokinase; CK-MB: creatine phosphokinase MB; COPD: chronic obstructive pulmonary disease; DYS: dyslipidemia; HBP: high blood pressure; HDL: high density lipoprotein; LDL: low density lipoprotein; LLA: lipid lowering agents; SE: standard error.

**Table 2:** Allele and genotype distribution of Del/Ins polymorphism by groups.

Genotype	HS	ACS			DM2		
	*n* (%)	*n* (%)	OR (CI)	*P*	*n* (%)	OR (CI)	*P*
Del/Del	60 (28)	27 (31)	—	—	16 (23)	—	—
Del/Ins	113 (53)	40 (47)	0.79 (0.44–1.40)	0.42	45 (65)	1.49 (0.78–2.86)	0.23
Ins/Ins	41 (19)	19 (22)	1.03 (0.51–2.09)	0.94	9 (12)	0.82 (0.33–2.04)	0.67
Allele							
Del	233 (54)	94 (55)	—	—	77 (55)	—	—
Ins	195 (46)	78 (45)	0.99 (0.69–1.41)	0.96	63 (45)	0.98 (0.66–1.43)	0.91

HS: healthy subjects, ACS: acute coronary syndrome, DM2: type 2 diabetes mellitus, OR: odd ratio, CI: confidence interval, Del: deletion, Ins: insertion.

**Table 3:** Clinical and demographic characteristics in the ACS and DM2 groups related to Del/Ins polymorphism of HLA-G by genetic models.

Phenotype	ACS (*n* = 86)	Codominant				*P*	Dominant			*P*	Recessive			*P*
		Del/Del (27)	Del/Ins (40)	Ins/Ins (19)	OR (CI)		Del/Del (27)	Del/Ins + Ins/Ins (59)	OR (CI)		Del/Del +Del /Ins (67)	Ins/Ins (19)	OR (CI)	
F/M	23/63	8/19	11/29	6/13	—	—	—	—	—	—	—	—	—	—
UA	12	3	7	2	0.94 (0.15–6.26)	1.00	3	9	1.44 (0.36–5.81)	0.75	10	2	0.67 (0.10–3.36)	0.63
STEMI	69	23	32	14	0.16 (0.05–0.59)	0.70	23	46	0.62 (0.18–2.10)	0.43	55	14	0.61 (0.18–2.02)	0.41
NSTEMI	5	1	1	3	4.88 (0.47–50.98)	0.29	1	4	1.89 (0.20–17.77)	1.00	2	3	6.09 (0.94–39.58)	0.06
DM2	36	12	19	5	0.45 (0.13–1.59)	0.23	12	24	0.86 (0.34–2.15)	0.74	31	5	0.40 (0.134–1.28)	0.12
HBP	45	10	24	11	2.34 (0.70–7.76)	0.23	10	35	**2.48 (0.97–6.33)**	**0.05**	34	11	1.34 (0.48–3.73)	0.58
DYS	21	6	12	3	0.66 (0.14–3.03)	0.72	6	15	1.19 (0.41–3.51)	1.00	18	3	0.51 (0.13–1.96)	0.32

Phenotype	DM2 (*n* = 70)	Codominant				*P*	Dominant			*P*	Recessive			*P*
		Del/Del (16)	Del/Ins (45)	Ins/Ins (9)	OR (CI)		Del/Del (16)	Del/Ins + Ins/Ins (54)	OR (CI)		Del/Del + Del/Ins (61)	Ins/Ins (9)	OR (CI)	

F/M	43/27	12/4	28/17	7/2	—	—	—	—	—	—	—	—	—	—
Overweight	35	6	23	6	1.93 (0.615–6.07)	0.23	6	29	1.93 (0.62–6.07)	0.40	29	6	2.21 (0.51–9.63)	0.28
HBP	34	6*	20	8^†^	**1.65 (0.15–1.60)** ^‡^	**0.02**	6	28	1.80 (0.57–5.64)	0.31	26	8	**3.03 (0.01–5.80)** ^‡^	**0.04**
Renal complications	7	1	6	0	0.54 (0.02–14.76)	1.00	1	6	1.88 (0.21–16.84)	1.00	7	0	0.38 (0.02–7.27)	0.28
Retinal complications	13	1	8	4	12 (1.07–134.11)	0.05	1	12	4.29 (0.51–35.83)	0.27	9	4	4.62 (1.04–20.57)	0.05
Dental complications	21	2	14	5	8.75 (1.2–63.428)	0.06	2	19	3.8 (0.78–18.51)	0.08	16	5	3.51 (0.84–14.73)	0.07
Cataracts	8	3	4	1	0.54 (0.05–6.14)	1.00	3	5	0.44 (0.09–2.10)	0.29	7	1	1.02 (0.11–9.39)	0.98

F: female, M: male, UA: unstable angina (use as reference with ACS types), STEMI: ST elevation myocardial infarction, NSTEMI: non-ST elevation myocardial infarction, DM2: type 2 diabetes mellitus, HBP: high blood pressure, DYS: dyslipidemia, COPD: chronic obstructive pulmonary disease, NS: not significant. *versus^†^: Del/Del versus Ins/Ins. ^‡^: OR corrected, Significant comparisons are highlighted in bold font.
